# Body mass as a result of psychological, lifestyle and genetic determinants. A pilot study involving overweight/obese and normal weight women in their early adulthood

**DOI:** 10.1371/journal.pone.0314942

**Published:** 2024-12-13

**Authors:** Małgorzata Obara-Gołębiowska, Katarzyna Eufemia Przybyłowicz, Anna Danielewicz, Tomasz Sawicki

**Affiliations:** 1 Faculty of Social Sciences, Department of Clinical, Developmental and Educational Psychology, University of Warmia and Mazury in Olsztyn, Olsztyn, Poland; 2 Faculty of Food Sciences, Department of Human Nutrition, University of Warmia and Mazury in Olsztyn, Olsztyn, Poland; District Head Quarter (DHQ) Hospital Charsadda / University of Peshawar, PAKISTAN

## Abstract

**Aim:**

The causes of obesity and overweight are complex and depend on mutually interrelated groups of lifestyle, psychological and genetic factors. Among 46 identified point mutations known within FTO gene, mutation SNP rs9939609 has the strongest effect on an increase in body weight. Therefore, the study aimed to assess psychological, lifestyle and genetic factors (expressed by the frequency of the FTO SNP rs-9939609 gene variant) and their association with body weight in young adult women.

**Methods:**

We genotyped FTO rs9939609 SNP in cheek swabs collected from 49 women aged 18–35, equally with and without overweight and obesity. Eating behaviour was defined based on the Questionnaire of Eating-Related Behaviors (QERB) and physical activity by the International Physical Activity Questionnaire (IPAQ). Classical anthropometric indices and the body composition analysis results measured by bioelectrical impedance were used to characterise nutritional status.

**Results:**

Overweight/obese participants had significantly higher body composition parameters than normal-weight participants, along with lower physical activity levels and more time spent sitting. Overweight/obese women presented significantly higher scores in emotional overeating, habitual overeating, and dietary restrictions, indicating more problematic eating behaviors compared to normal-weight individuals. No significant differences were observed in BMI, lifestyle factors, or eating behaviors across FTO gene variants (AA, AT, and TT). However, the TT and AT FTO gene variant groups showed significant positive correlations between habitual overeating and key measures of body weight (BMI, WC, and FM). In contrast, the AA variant group exhibited fewer associations between psychological and lifestyle factors with body weight.

**Conclusions:**

Psychological and lifestyle factors, particularly overeating behaviors, were more strongly associated with increased body weight in women with the TT and AT variants of the FTO SNP rs-9939609 gene, highlighting the potential influence of genetic predisposition on eating habits and weight status in this population. Proper eating habits and high physical activity play an important role in preventing overweight and obesity regardless of the genotype that poses a potential risk of weight gain. The study’s findings bring practical implications for health education and health psychology.

## Introduction

Causes of obesity and overweight are complex and depend on mutually interrelated factors [1–3]. The first group of factors are changes in modern civilisation determining human lifestyles [4,5]. This group of factors mainly includes poor nutrition and low levels of physical activity [4,6–8]. Other factors related to the occurrence of obesity are psychological ones [9]. According to psychological theories, they particularly include disturbances in self-regulation of eating [10,11]. The process of self-regulation permits the adjustment of one’s own reactions to internal or external expectations and standards, allowing them to be both an expression of momentary needs and longer-term priority goals [12]. Two categories of self-regulation disorders leading to improper eating behaviour are distinguished. The first one is insufficient regulation, where a person does not have sufficient control over themselves. Insufficient regulation can lead to habitual and emotional overeating [13]. The second category of self-regulation disorders is improper regulation, where a person attempts to control themselves and the reality, but eventually does not obtain the desired result [11]. Improper regulation particularly involves extreme/inappropriate forms of diet restrictions, also resulting in eventual loss of control overeating [13,14].

There are also genetic factors linked to the cause of obesity [15,16]. FTO gene is related to the pathogenesis of obesity [17]. FTO gene is located on the long arm of chromosome 16 (16q12.2) and is highly polymorphic [18–20]. It codes protein with a function of demethylase demethylation of nucleic acids depending on 2-oxoglutarate. The enzyme participates in the repair of the DNA, metabolism of fatty acids, and post-translation modifications [19,20]. The gene is expressed in various tissues, particularly in the brain, in the centre of appetite control and energy expenditure in the hypothalamus. Although the functioning mechanism of FTO gene has not been thoroughly investigated yet, it probably participates in the control of food intake and determines dietary preferences [21]. It also influences the regulation of energy expenditure. It appears that expression of genetic information coded by certain alleles of FTO gene is correlated with an increase in body weight [22,23]. The findings also suggest that the FTO gene may affect the amount of body fat, and thus the size of BMI also by affecting feelings of satiety and appetite [24].

Among 46 identified point mutations within FTO gene, mutation SNP rs9939609 has the strongest effect on an increase in body weight and occurrence of overweight and obesity (including their consequences, namely type 2 diabetes) [25]. This allele is common in the European population and considered as one of the primary factors of genetically determined occurrence of obesity [20]. Allele (A) is known not to be related to body weight at the moment of birth, but it contributes to the development of obesity from early childhood [26]. In homozygotes with allele (A), a higher BMI value is observed than in persons without the allele in the genotype [20]. A direct correlation has also been determined between the occurrence of allele (A) in the genotype and occurrence of type 2 diabetes. The correlation involves the effect of the variant of FTO gene on individual body mass [27–29]. The study also analyses the correlation between the presence of the polymorphism of the FTO gene and mortality. The results indicate that the allele (A) may contribute to increased mortality by promoting diseases associated with associated with obesity [30,31].

As described above according to multiple studies, the FTO gene is one of the genes determining the complex genetic basis of obesity and its consequences metabolic consequences.

However, there is a lack of research that considers the interplay of psychological, lifestyle and genetic factors on body mass. For this reason, learning about the genetic aspects of obesity and how they relate to other factors that cause weight gain perhaps soon will allow us to for a more effective fight against this serious scourge of our times. Studies showing the correlations between psychological factors influencing lifestyle, FTO gene variants, and body mass, however, are scarce. Moreover, results of the studies are ambiguous.

The proposed study aimed to assess psychological, lifestyle and genetic factors (expressed by the frequency of the FTO SNP rs-9939609 gene variant) and their association with the body weight in young adult women. We decided to study the prevalence of A and T alleles in 2 groups of people (normal weight and overweight) and compare them in terms of eating behaviour (emotional overeating, habitual overeating, diet restrictions) and physical activity. The inclusion of such a group of people in the study is dictated by the fact that the psychophysical health and nutritional status of women of reproductive age affects the metabolic programming of future generations. In addition, the study is a pilot that is the basis for further research involving groups of people of different ages and genders.

## Material and methods

### Participants

The study included 2 equal groups of young adult women aged 18–35 years: normal weight (18.5 < BMI < 25 kg/m2), and overweight and obese (BMI >25 kg/m^2^), totalling 50 subjects. Participants were female students of the University of Warmia and Mazury in Olsztyn recruited from March to September 2020. The inclusion criteria were: female sex, age 18–35 years, normal weight or indicating overweight or obesity. The exclusion criteria for the study were failure to meet the basic inclusion criteria (above), period of pregnancy, lactation, restriction of legal activities, lack of consent to participate in the study, and limitation of sanity due to mental retardation, severe mental disorders, or addictions.

Based on the previous own studies [32] and the available literature, we assumed that the eating behaviours score of young women with obesity is 15.8 ± 6.4, and that normal-weight women have about 30% lower QERB scores. Assuming a given 95% confidence level and 80% power, the required sample size is 62 subjects, 31 each in the normal weight and overweight and obese women groups. As the study was a pilot, as many participants as possible were recruited due to limited funding to carry it out. Due to missing data, it was necessary to exclude one person from further analysis.

The study was approved by the Ethics Committee for Scientific Research of University of Warmia and Mazury, Olsztyn, Poland. Sensitive data were collected in an “as-less-as-necessary” manner and are protected and managed by the principal investigator and data manager. Only authorised collaborators have an access to data. Personal data are processed in accordance with valid laws and regulations and at the units of University of Warmia and Mazury in Olsztyn, personal data is protected at all levels. All participants gave written consent to participate in the study.

### Anthropometric measurements

Anthropometric measurements of body weight (kg) and height (m) were carried out in light clothing and barefoot, with the use electronic scale and stadiometer, respectively. Waist circumference was measured using an non-elastic tape and expressed in metres. BMI was calculated by dividing body mass by height squared. an interpret with the criteria of WHO as underweight (BMI ≤ 18.5 kg/m^2^), normal body weight (18.5 ≤ BMI < 25 kg/m^2^, and overweight and obesity (BMI ≥ 25 kg/m^2^). To assess body fat content and visceral adipose tissue volume by bioelectrical impedance the body composition segment analyser were used (SECA 515mBCA, Allers, Hamburg, Germany).

### Eating behaviors

Data were collected on self-regulation of eating in terms of emotional and habitual overeating and dietary restrictions. The diagnostic method used was the Questionnaire of Eating-Related Behaviors–QERB [13]. It permits measurement of behaviour related to eating, including diagnosis of eating disorders, prediction of tendencies to gain weight, and determination of causes of excessive eating leading to overweight or obesity. Each positive answers was awarded one point, except five questions where one point was awarded for a ‘no’ answer. Overall scores range from 0 to 30 while individual subscale scores range from 0 to10. It permits the determination of whether loss of control overeating, i.e. overeating, is of habitual or emotional character, or is the effect of excessive and improper diet restrictions. The higher the score on the whole scale and on individual subscales, the more abnormal the eating behaviour, which is a risk of developing eating disorders or excessive body weight. Reliability analysis for Questionnaire of Eating-Related Behaviors: 0.671—overall QERB; 0,632—habitual overeating; 0.560—emotional overeating; 0.560—diet restrictions.

### Physical and sedentary activity

To assess the level of physical activity the Polish adaptation of the International Physical Activity Questionnaire (IPAQ) was used [33,34]. When answering, participants specified the time and frequency of physical activity in the work-related, transport-related, domestic and gardening (yard), and leisure-time domains. They determined how many minutes and days in the last week before the survey they had spent on these activities. Physical activity was expressed as a sum of all activities (MET (metabolic equivalent of task)-min/week). The sum of the average time per week spent on sitting (at work and home, and while driving) expressed the sedentary time (hour/week).

### Genetic testing

A swab was taken from the inside of the cheek of each person included in the study (50 people, 2 groups of 25 people each). The collected genetic material was transferred to a specialized laboratory, where the genotypes at the locus containing the FTO gene in the subjects were determined. The order was carried out according to the internal procedures of the Genomed laboratory. According to the information provided by the laboratory, the procedure was as follows. The PCR product was purified from excess primers or dNTp remaining after the PCR reaction using the Exo-Sap kit (according to the manufacturer’s instructions). Then, 3ul of BigDye ™ Terminator v3.1 Ready Reaction Mix, 1ul of BigDye ™ Terminator v 1.1. and v3.1 5X sequencing buffer, 5 pmol of the appropriate primer and 25–50 ng of DNA template were added to each sequencing reaction. The whole was resuspended in a final volume of 10 ul. The sequencing reaction was carried out as follows, in 100ul PCR tubes. Incubation at 96°C for 1 min as the initial denaturation step, followed by 25 cycles of 96°C for 10 sec, 54°C for 5 sec. and 60°C for 4 min. Before purification, the reaction mixture was incubated at 4°C. Purified reaction products were separated by electrophoresis on a 3730xl DNA analyzer according to the manufacturer’s references (Thermofisher). For further comparisons between individuals, the FTO SNP rs-9939609 gene variant was divided into AA homozygotes (mutation in both alleles), TT homozygotes without mutations in both gene alleles) and AT heterozygotes (with a mutation in one gene allele).

### Statistical analysis

The normality of the distribution of the variables was analysed using the Shapiro-Wilk test. Since not all variables had a normal distribution, qualitative variables were presented as median and 25th and 75th quartile range. The Mann-Whitney U test was used to analyse differences between two groups, and the Kruskal Wallis test Dunn’s post-hoc test was used to analyse differences between multiple groups. A correlation analysis was also carried out using Pearson’s test. Qualitative variables were presented as counts and percentages, and their distribution between groups was assessed using Pearson’s chi-square test. In all analyses, the level of statistical significance was set at p<0.05. Statistical analysis was performed with TIBCO® Statistica™ ver. 13.3 (TIBCO Software Inc., Tulsa, OK).

## Results

The data presented in Table 1 anthropometrics, lifestyle factors, eating behaviours and FTO gene variant among overweight and normal weight groups. The sample consisted of 49 participants with a median age of 21 years. In the overweight/obese group compared to the normal-weight group were observed significantly higher weight (85.8 kg vs. 58.3 kg, p < 0.001), BMI (30.5 kg/m^2^ vs. 21.5 kg/m^2^, p < 0.001), waist circumference (0.9 m vs. 0.7 m; p < 0.001) and body fat content (40.3% vs. 28.7%; p < 0.001). Visceral adipose tissue volume was also elevated in the overweight/obese group (1.4 L vs. 0.5 L, p < 0.001). Total physical activity measured in MET-min/week was significantly lower in the overweight/obese group (4739 MET-min/week) than in the normal-weight group (7878 MET-min/week, p = 0.042). However, time spent sitting was significantly higher in the overweight/obese group (4.2 hours/day) than in the normal-weight group (2.2 hours/day, p = 0.017). The total QERB score was significantly higher in the overweight/obese group (16 vs. 10, p < 0.001), indicating more problematic eating behaviors. Subcomponents of the QERB revealed significantly higher scores in the overweight/obese group for emotional overeating (5 vs. 4, p = 0.016), habitual overeating (6 vs. 3, p < 0.001), and dietary restrictions (5 vs. 3, p = 0.041). The distribution of the FTO rs-9939609 gene variant alleles (TT, AT, AA) did not differ significantly between the groups (p = 0.675).

**Table 1:** Anthropometrics, lifestyle factors, eating behaviours and FTO gene variant among overweight and normal weight groups.

Characteristic	Total	Overweight and obesity	Normal weight	p
*n*	49	24	25	
Age, y	21 (20; 22)	20.5 (20; 25)	21 (20; 21)	0.556
Weight, kg	69.8 (58.3; 85.4)	85.8 (77.5; 95)	58.3 (54.4; 62.6)	<0.001
Height, m	1.7 (1.6; 1.7)	1.7 (1.6; 1.7)	1.7 (1.6; 1.7)	0.458
BMI, kg/m^2^	24.4 (21.5; 30.4)	30.5 (27; 35.4)	21.5 (20.3; 22.7)	<0.001
WC, m	0.9 (0.7; 0.9)	0.9 (0.9; 1)	0.7 (0.7; 0.8)	<0.001
FM, %	35.8 (28.7; 39.9)	40.3 (38.7; 44.9)	28.7 (25.5; 30.9)	<0.001
VAT, l	0.9 (0.5; 1.3)	1.4 (1.2; 2.2)	0.5 (0.4; 0.7)	<0.001
Physical activity, MET-min/wk	5204 (2493.3; 8223.1)	4739 (2158.8; 7028)	7878 (3594; 9574)	0.042
Physical activity level^†^				
Low	2 (4.1)	2 (8.4)	0 (0.0)	0.329
Medium	10 (20.4)	5 (20.8)	5 (20.0)	
High	37 (75.5)	17 (70.8)	20 (80.0)	
Time spent sitting, h	3.8 (1.9; 5.2)	4.2 (3.4; 5.8)	2.2 (1.6; 4.1)	0.017
QERB total, points	13 (8; 17)	16 (13; 19)	10 (7; 13)	<0.001
Emotional overeating	4 (3; 6)	5 (3; 7.5)	4 (2; 4)	0.016
Habitual overeating	5 (3; 7)	6 (5; 8)	3 (2; 5)	<0.001
Dietary restrictions	4 (2; 5)	5 (3; 6)	3 (2; 5)	<0.041
FTO rs-9939609 gene variant^†^				
TT	15 (30.6)	8 (33.3)	7 (28.0)	0.675
AT	21 (42,7)	11 (45.8)	10 (40.0)	
AA	13 (26.5)	5 (20.8)	8 (32.0)	

Quantitative data are presented as median and range of 25th-75th quartile and ^†^qualitative data are presented as number and %. QERB—Questionnaire of Eating-Related Behaviors, BMI–body mass index, WC–waist circumference, FM–fat mass, VAT–visceral adipose tissue, MET–metabolic equivalent of tasks. TT, AT, AA–allele variants of FTO SNP rs-9939609 gene.

p≤0.05 statistical significance (U Mann-Whitney test for qualitative variables or Pearsons’ chi-square test for quantitative variables).

Table 2 compares psychological and lifestyle factors across subgroups of the FTO SNP rs-9939609 gene variant (AA, AT, and TT). There were no statistically significant differences in anthropometric measures, physical activity, sedentary behaviour, or eating behaviours across the FTO SNP rs-9939609 gene variants (AA, AT, and TT).

**Table 2:** Differences between psychological and lifestyle factors through the subgroups of FTO SNP rs-9939609 gene variant.

	FTO SNP rs-9939609 gene variant			p
	AA	AT	TT	
*n*	13	21	15	
Age, y	20 (20; 21)	21 (20; 27)	21 (20; 23)	0.697
Weight, kg	66.5 (61.5; 83.7)	71.2 (58.1; 85.4)	75.1 (54.4; 89.9)	0.912
Height, m	1.7 (1.6; 1.7)	1.7 (1.6; 1.7)	1.7 (1.6; 1.7)	0.578
BMI, kg/m^2^	23.6 (21.7; 29.3)	25.9 (21.1; 30.4)	25.5 (20.3; 30.8)	0.922
WC, m	0.8 (0.8; 0.9)	0.9 (0.7; 1)	0.9 (0.7; 0.9)	0.891
FM, %	33.2 (29.9; 39.2)	37 (29.5; 41.1)	37.3 (24.8; 40.7)	0.778
VAT, l	1.1 (0.4; 1.3)	0.9 (0.5; 1.5)	0.8 (0.4; 1.2)	0.670
Physical activity, MET-min/wk	4764 (2940; 7986)	5393.5 (2222; 8223.1)	4725 (2225; 9680)	0.989
Time spent sitting, h	2.2 (1.5; 4.3)	4.1 (2.2; 6)	3.5 (1.7; 5.6)	0.142
QERB total	12 (8; 19)	13 (10; 17)	15 (8; 17)	0.954
Emotional overeating	3 (2; 4)	4 (3; 6)	4 (2; 7)	0.377
Habitual overeating	4 (3; 7)	5 (3; 6)	6 (3; 7)	0.919
Dietary restrictions	5 (3; 7)	4 (2; 5)	4 (2; 6)	0.669

Quantitative data are presented as median and range of 25th-75th quartile. QERB—Questionnaire of Eating-Related Behaviors, BMI–body mass index, WC–waist circumference, FM–fat mass, VAT–visceral adipose tissue, MET–metabolic equivalent of tasks. TT, AT, AA–allele variants of FTO SNP rs-9939609 gene.

p≤0.05 statistical significance (Kruskal-Wallis test with Dunn’s post-hoc test).

Table 3 presents the correlation analysis between psychological and lifestyle factors among different subgroups (TT, AT, AA) of the FTO SNP rs-9939609 gene variant. Among participants with TT variant of FTO gene habitual overeating showed significant positive correlations with weight (r = 0.679, p ≤ 0.01), BMI (r = 0.681, p ≤ 0.01), waist circumference (WC, r = 0.596, p ≤ 0.05), fat mass percentage (FM, r = 0.723, p ≤ 0.01), and time spent sitting (r = 0.655, p ≤ 0.01). A significant negative correlation was observed with physical activity (r = -0.568, p ≤ 0.05). The total QERB score demonstrated significant positive correlations with weight (r = 0.631, p ≤ 0.01), BMI (r = 0.646, p ≤ 0.01), WC (r = 0.529, p ≤ 0.05), and FM (r = 0.619, p ≤ 0.05). A negative correlation was found between height and dietary restrictions (r = -0.544, p ≤ 0.05).

**Table 3:** Correlation between psychological and lifestyle factors through the subgroups of FTO SNP rs-9939609 gene variant.

	Questionnaire of Eating-Related Behaviors			
	Emotional overeating	Habitual overeating	Dietary restrictions	QERB total
TT				
Weight, kg	0.510	0.679**	0.239	0.631**
Height, m	0.181	-0.103	-0.544*	-0.172
BMI, kg/m^2^	0.413	0.681**	0.393	0.646**
WC, m	0.375	0.596*	0.235	0.529*
FM, %	0.414	0.723**	0.277	0.619*
VAT, l	0.389	0.483	0.243	0.490
Physical activity, MET-min/wk	-0.288	-0.568*	-0.254	-0.483
Time spent sitting, h	0.414	0.655**	-0.108	0.438
AT				
Weight, kg	0.360	0.643**	0.719***	0.692***
Height, m	0.010	-0.093	0.203	0.045
BMI, kg/m^2^	0.376	0.662***	0.645**	0.678***
WC, m	0.336	0.528*	0.222	0.444*
FM, %	0.351	0.700***	0.574**	0.655***
VAT, l	0.385	0.520*	0.511*	0.573**
Physical activity, MET-min/wk	0.031	-0.141	-0.025	-0.052
Time spent sitting, h	-0.162	-0.118	-0.001	-0.118
AA				
Weight, kg	0.122	0.086	0.214	0.173
Height, m	-0.165	0.021	0.028	-0.046
BMI, kg/m^2^	0.184	0.098	0.260	0.221
WC, m	0.317	0.299	0.335	0.388
FM, %	0.275	0.224	0.314	0.332
VAT, l	0.305	0.271	0.315	0.364
Physical activity, MET-min/wk	-0.075	0.288	0.307	0.215
Time spent sitting, h	0.627*	0.373	-0.020	0.396

Data are presented as correlation coefficient (r). QERB—Questionnaire of Eating-Related Behaviors, BMI–body mass index, WC–waist circumference, FM–fat mass, VAT–visceral adipose tissue, MET–metabolic equivalent of tasks. TT, AT, AA–allele variants of FTO SNP rs-9939609 gene.

*p≤0.05

**p≤0.01

***p≤0.001 (Pearson’s correlation test).

Participants with AT variant of FTO gene showed strong positive correlations of habitual overeating and weight (r = 0.643, p ≤ 0.01), BMI (r = 0.662, p ≤ 0.001), WC (r = 0.528, p ≤ 0.05), FM (r = 0.700, p ≤ 0.001), and VAT (r = 0.520, p ≤ 0.05). Dietary restrictions correlated positively with weight (r = 0.719, p ≤ 0.001), BMI (r = 0.645, p ≤ 0.01), and FM (r = 0.574, p ≤ 0.01). The total QERB score was significantly correlated with weight (r = 0.692, p ≤ 0.001), BMI (r = 0.678, p ≤ 0.001), FM (r = 0.655, p ≤ 0.001), and VAT (r = 0.573, p ≤ 0.01).

Through the participants with AA variant of FTO gene time spent sitting showed a significant positive correlation with emotional overeating (r = 0.627, p ≤ 0.05), but other correlations were not statistically significant.

## Discussion

Obesity is expressed in body weight index. However, like any quantitative phenotypic trait, it can be modified by environmental factors significantly correlated with a person’s lifestyle. Also, the psychological condition of a person, constituting a resultant of the effect of the environment and biology, may interact with the consequences of the expression of genetic information coded by the alleles. The purpose of the study presented was to try to find an answer to the question about the interdependencies between psychological, lifestyle and genetic factors- expression of genetic information coded by variant of FTO gene (presence or lack of allele A) contributing to weight gain, in young adult women aged 18–35. This question was answered by examining overweight/obese and normal-weight women for the presence of SNP rs9939609 mutations of the FTO gene they had on their chromosomes.

The study compared overweight and normal-weight individuals for the severity of abnormal eating behaviors (Table 1). The overall QERB questionnaire score was found to be higher among overweight women. This indicates an overall higher severity of abnormal eating-related self-regulatory strategies that increase the likelihood of excessive weight or eating disorders. However, an analysis of differences between the subscales indicated significant intergroup differences on only one subscale of the QERB questionnaire. It turned out that overweight subjects were significantly more likely than thin subjects to manifest disruptions in self-regulation of eating through habitual overeating. In contrast, there was no significantly higher intensity of emotional overeating or the use of maladaptive eating restriction strategies that could potentially lead to subsequent disinhibition and overeating. Among obese individuals, the overall questionnaire score and the score on the habitual overeating subscale indicate moderate severity of the variables, compared to a group of thin individuals who scored low in this area. A number of studies analyzing the self-regulation of eating in overweight and obese individuals note the significantly higher severity of maladaptive self-regulatory strategies related to eating in overweight individuals compared to normal weight individuals [20,25,26,29]. In contrast, comparisons of individuals differentiated by their genotype indicate no differences in eating behavior (Table 2). It should also be mentioned that in the compared groups of overweight and normal-weight women, the prevalence of the A and T alleles was similar (Table 2). The results suggest that in the groups compared, possession of a gene that represents a potential risk for overeating and weight gain did not matter. In contrast, other studies show that persons that have homozygotes with two alleles (A) in the genotype display higher body weight, and considerably higher susceptibility to obesity. In contrast to persons not having the (A) allele- so called homozygotes (TT), they are more frequently observed to have tendencies towards improper dietary habits–including choosing meals with substantial amounts of fat [23]. It should be mentioned that not only homozygotes (AA) but also heterozygotes (AT)-people who have one allele of the gene with the SNP rs9939609 mutation-are at greater risk of increased body weight than homozygotes (TT) [35,36]. The presence of the allele A in the genotype shows a strong correlation with increased body circumference in the hips, and increased body weight [31,37,38].

The study also compared groups differentiated by weight (Table 1) and genotype (Table 2) by level of physical activity. Significant differences were observed in the overall level of physical activity and sitting time among those differing by body weight, but not genes possessed. A significantly higher percentage of people with high physical activity was observed among those with normal body weight. As is well known, high physical activity is a protective factor in the prevention of weight gain [1,2,39,40].

In spite of the promising results, some limitations of the study carried out should be considered. The group of study subjects was not very diverse. The study included young, college-educated, female participants. It is known that the level of education, age and gender can affect the results obtained. Among other things, a higher level of education and female gender are variables positively correlated with the level of education and health practice [3,4]. In addition, the expression of presence or absence of SNP rs9939609 mutation of the FTO gene may be dependent on the age of the patient in relation to gender [34]. In the study conducted, other factors of a physiological nature that may affect one’s body weight and eating habits were not controlled. Also, examining the body mass composition of the subjects could bring a number of inspiring conclusions to the results. For this reason, future research on larger groups of people differentiated by gender, age and education level would also make it possible to create nutritional profiles using measurement tools used in dietetics and psychology. It is worth emphasizing, however, that the presented study was a pilot study. The results obtained have both practical implications in the treatment of patients with excessive body weight and theoretical implications that provide a basis for conducting extended and more in-depth research on the subject.

Given that many people believe that overweight and obese patients can treat themselves, this may lead to a growing epidemic of overweight and obesity. We must remember that weight disorders are a complex multi-causal disease where environmental, metabolic, endocrine and psychological causes may overlap with genetic predisposition. The research carried out, despite its limited genetic diagnosis, points to the development of more individualised education and application programs to reduce the incidence of overweight and obesity in relation to genetic determinants. We know for sure that in order to effectively deal with overweight and obese patients, we need a multidisciplinary treatment team, including a medical psychologist and a dietician. To develop psychological support programs for these individuals, which in the long term will select an individual treatment plan that takes into account all the determinants of weight disorders. Psychological therapy to change the patient’s relationship with food intake is a major challenge for both the patient and the dietitian, but also essential in regulating the disorder. The mechanisms of the diet which might show promise as an aid to manage mental and neurodegenerative disorders. Include the modulation of the gut microbiota and the gut-brain axis, the regulation of inflammatory pathways, a reduction in oxidative stress, and the promotion of neuroplasticity [40]. It is also notable here that, in the light of recent research into the brain, it would be very interesting to analyse neuroanatomy and brain function also in relation to diet, which is undoubtedly a future-oriented research direction. Bearing in mind the recent study by Sam Vickery et al. [41] reports that furthering our understanding about specific neurobiological influences on spatial patterns of brain aging may provide insight into the brain changes in healthy aging and possible diagnostic markers for clinical outcomes. Authors say, it would be interesting to look at age-related changes in the connections between brain regions and gene expression profiles [41] research conducting in this way may allow the development of systemic psychological support programs with high patient impact.

## Conclusion

The proposed project focused on lifestyle and psychological factors of high importance in the diagnosis and psychotherapy of persons with excessive body weight. They included physical activity and self-regulation-induced eating behaviour such as: emotional overeating, habitual overeating, diet restrictions. Conclusions from the study draw attention to the special role of proper eating habits and high physical activity in the prevention of overweight and obesity regardless of the genotype that poses a potential risk of weight gain. However, due to the limitations of the study groups, conclusions should be drawn with caution.

The issue discussed in the presented research project was of interdisciplinary character, exceeding the competences of a single field of science. The project combined research techniques characteristic of social and environmental sciences. The study presented constitutes contribution to the development of the fields of such sciences as health education, health promotion and health psychology in reference to the development, and prevention of overweight.

The study’s main limitation is the small sample size, which does not allow for advanced multivariate statistical procedures. In future studies, we plan to expand the research to different age groups of both women and men, which will enable the observation of age-related disorders and the scale of genetic influence in relation to age. Additionally, analysing dietary habits, the microbiome, and lifestyle factors such as sleep duration and quality will provide a multi-layered interpretation of the results. Understanding the mechanisms predisposing to and participating in the development of body mass disorders is essential if we are to effectively prevent and treat them.

## Supporting information

S1 Dataset Raw study data used in analyses. (XLSX)
